# Targeting MLL Methyltransferases Enhances the Antitumor Effects of PI3K Inhibition in Hormone Receptor–positive Breast Cancer

**DOI:** 10.1158/2767-9764.CRC-22-0158

**Published:** 2022-12-06

**Authors:** Robert B. Jones, Jonathan Farhi, Miranda Adams, Kiran K. Parwani, Garrett W. Cooper, Milica Zecevic, Richard S. Lee, Andrew L. Hong, Jennifer M. Spangle

**Affiliations:** 1 Department of Radiation Oncology, Winship Cancer Institute of Emory University, Atlanta, Georgia.; 2 Cancer Biology Graduate Program, Emory University School of Medicine, Atlanta, Georgia.; 3 Genetics and Molecular Biology Graduate Program, Emory University School of Medicine, Atlanta, Georgia.; 4 Department of Biology, Emory University, Atlanta, Georgia.; 5 Department of Pediatrics, Emory University School of Medicine, Atlanta, Georgia.

## Abstract

The high frequency of aberrant PI3K pathway activation in hormone receptor–positive (HR^+^) breast cancer has led to the development, clinical testing, and approval of the p110α-selective PI3K inhibitor alpelisib. The limited clinical efficacy of alpelisib and other PI3K inhibitors is partially attributed to the functional antagonism between PI3K and estrogen receptor (ER) signaling, which is mitigated via combined PI3K inhibition and endocrine therapy. We and others have previously demonstrated chromatin-associated mechanisms by which PI3K supports cancer development and antagonizes ER signaling through the modulation of the H3K4 methylation axis, inhibition of KDM5A promoter H3K4 demethylation and KMT2D/MLL4-directed enhancer H3K4 methylation. Here we show that inhibition of the H3K4 histone methyltransferase MLL1 in combination with PI3K inhibition impairs HR^+^ breast cancer clonogenicity and cell proliferation. While combined PI3K/MLL1 inhibition reduces PI3K/AKT signaling and H3K4 methylation, MLL1 inhibition increases PI3K/AKT signaling through the dysregulation of gene expression associated with AKT activation. These data reveal a feedback loop between MLL1 and AKT whereby MLL1 inhibition reactivates AKT. We show that combined PI3K and MLL1 inhibition synergizes to cause cell death in *in vitro* and *in vivo* models of HR^+^ breast cancer, which is enhanced by the additional genetic ablation of the H3K4 methyltransferase and AKT target KMT2D/MLL4. Together, our data provide evidence of a feedback mechanism connecting histone methylation with AKT and may support the preclinical development and testing of pan-MLL inhibitors.

**Significance:**

Here the authors leverage PI3K/AKT-driven chromatin modification to identify histone methyltransferases as a therapeutic target. Dual PI3K and MLL inhibition synergize to reduce clonogenicity and cell proliferation, and promote *in vivo* tumor regression. These findings suggest patients with PIK3CA-mutant, HR^+^ breast cancer may derive clinical benefit from combined PI3K/MLL inhibition.

## Introduction

Aberrant activation of the PI3K pathway, including mutational activation of the PIK3CA gene that encodes the lipid kinase p110α, is a common event in hormone receptor–positive (HR^+^) cancers (1). The high frequency of PI3K activation, coupled with its essential roles in cell growth, proliferation, and survival, is consistent with preclinical and clinical activity of the p110α-selective PI3K inhibitor alpelisib in HR^+^ breast cancer. As of 2019, alpelisib is FDA approved for the treatment of metastatic PIK3CA-mutant, HR^+^ breast cancers in combination with the estrogen receptor (ER) antagonist fulvestrant (2). Despite this success, preclinical and clinical studies utilizing PI3K inhibitors including alpelisib lack efficacy when used as monotherapies, and resistance remains a common occurrence. Thus, achieving an efficacious response to PI3K inhibition remains an unmet clinical need.

Histones serve an integral role downstream of signaling pathways by supporting posttranslational modifications (PTM) including phosphorylation, acetylation, and methylation (3), which contribute to chromatin state and gene expression. The PI3K effector AKT modulates the activity of enzymes responsible for the reading, writing, and erasure of histone PTMs, which may support tumorigenesis. AKT phosphorylates the H3K27 histone methyltransferase EZH2 (4) and the histone acetyltransferase p300 (5). AKT phosphorylation of the H3K4 demethylase KDM5A (6) and the H3K4 methyltransferase KMT2D (also referred to as MLL2/4; ref. 7) in breast cancer has been shown to modulate promoter H3K4me3 and enhancer H3K4me1, respectively. Dysregulation of H3K4 methylation enhances gene expression to support pro-oncogenic growth and therapeutic resistance, indicating that H3K4 methylation and associated epigenomic regulation may be a critical downstream effector of aberrant PI3K/AKT signaling in breast and other cancers. Clinical studies demonstrate that aggressive breast and other cancers are characterized by elevated H3K4 methylation and that H3K4 trimethylation (H3K4me3) is an indicator of poor patient prognosis (8–10).

While six encoded macromolecular MLL/COMPASS complexes that direct cellular H3K4 methylation are encoded in the human genome (11), AKT-mediated phosphorylation has been shown to regulate the activity of KMT2D/MLL4 (7), which primarily monomethylates enhancers. AKT indirectly increases promoter H3K4me3 through the inactivation of the H3K4 demethylase KDM5A (6), and when coupled with AKT phosphorylation of KMT2D/MLL4, these studies suggest at least two mechanisms by which PI3K/AKT signaling modulates H3K4-driven epigenetic plasticity to contribute to cancer development and therapeutic response. The activity of KDM5A is antagonized by the MLL1 COMPASS complex, which acts on promoters to trimethylate H3K4. Here, we demonstrate that combined therapeutic targeting of PI3K/AKT and the H3K4 methyltransferase MLL1 decreases H3K4me3 to reduce transformative properties of human breast cancer cell lines and tumor growth in murine models, which is further enhanced following genetic loss of KMT2D/MLL4. Collectively, these data suggest that the therapeutic targeting of MLL1- and KMT2D/MLL4-directed H3K4 methylation may be beneficial HR^+^, PIK3CA-mutated, breast cancer. We also uncover a previously uncharacterized feedback loop between MLL1 and AKT, which may suggest PI3K effector activation as a possible mechanism of MLL1 inhibitor resistance.

## Materials and Methods

### Cell Lines, Inhibitors, siRNA Reagents

Authenticated MCF7, T47D, MCF10A, HCC1500, and 293T cells were purchased from ATCC and used for experiments within the first 10 passages. Cultures were checked for *Mycoplasma* every 6 months (Lonza) and cells maintained at 37°C and 5% CO_2_ in RPMI or DMEM + 10% FBS and 1% Pen-Strep. GDC-0941, BYL719, GDC-0032, and GDC-0068 were purchased from Selleck, and MI-136, MI-503, and MM-102 were purchased from Cayman. siRNAs (Silencer Select) were purchased from Invitrogen (Negative control #1, 4390843; siKMT2D, s528766).

### Cell/Tumor Lysis and Immunoblotting

Cells were lysed in immunoprecipitation buffer [20 mmol/L TrisHCl (pH 7.5), 150 mmol/L NaCl, 5 mmol/L MgCl_2_, 1% NP-40] and tumors were lysed in RIPA buffer [10 mmol/L TrisHCl (pH 8.0), 1 mmol/L Ethylenediaminetetraacetic acid (EDTA), 0.1 mmol/L Egtazic acid (EGTA), 1% TritonX-100, 0.1% Sodium Deoxycholate, 0.1% SDS, 140 mmol/L NaCl], supplemented with protease and phosphatase inhibitors. Histones were acid extracted from cells by lysing in triton extraction buffer (TEB; PBS, 0.5% Triton X-100) supplemented with protease inhibitors. Tumors were sonicated (Diagenode) prior to lysis or histone extraction. Cell and tumor lysates were centrifuged 6,500 × *g* and histones were acid extracted from the resulting pellet with 1:1 TEB:0.8 mol/L HCl. Histones were centrifuged and the supernatant precipitated with the addition of an equal volume of 50% tricarboxylic acid, then 12,000 × *g* centrifugation. Histones were washed one time in ice-cold 0.3 mol/L HCl in acetone and two times ice cold in 100% acetone before drying and resuspended in 20 mmol/L Tris-HCl (pH 8.0), supplemented with protease inhibitors. Whole-cell lysate or acid-extracted proteins were separated using SDS-PAGE. Proteins were transferred to nitrocellulose membranes and blocked in tris-buffered saline + 0.1% Tween-20 + 5% milk. Proteins of interest were visualized and quantified after primary antibody incubation (Odyssey, LI-COR). Primary antibodies used were as follows: AKT (Cell Signaling Technology 9272), AKTS473 (Cell Signaling Technology 9271), S6 (Cell Signaling Technology 2217), S6S240/44 (Cell Signaling Technology 2215), LIN28A (Cell Signaling Technology 3978), β-actin (Millipore-Sigma MAB1501), KMT2D/MLL4 (Millipore-Sigma ABE1867), PRUNE2 (Millipore-Sigma HPA031079), H3 (Abcam 1791), H3K4me3 (Abcam 8580), H3K4me2 (Abcam 32356), and H3K4me (Abcam 8895).

### Cell Viability, Apoptosis, and Clonogenic Assays

For resazurin viability assays, 3,000 cells/well were seeded in 96-well plates. The next day, serially diluted inhibitors were added and incubated for 5 days, after which resazurin (Sigma R7017) was added to a final concentration of 0.1 mg/mL and incubated for 5 hours prior to measuring the excitation and/or emission (544/590; Biotek). Cells were reverse transfected (Lipofectamine 3000) with siRNAs according to manufacturer's instructions (Invitrogen) where indicated. To assay for apoptosis, cells were seeded and treated with the indicated inhibitors for 5 days, after which cells were detached using accutase (BioLegend) and stained using Annexin V/propidium iodide following manufacturer's protocol (BioLegend). Cells were kept in the dark until assessment and were analyzed by flow cytometer (BDFACSymphony A3) within 1 hour. To measure cell clonogenicity, 500 cells/well were seeded in a 6-well plate, and treated the following day with the designated inhibitors, and incubated for a total of 19 days with media and inhibitor change every 3–4 days, after which cells were fixed and crystal violet stained. Plates were imaged, destained, and crystal violet quantitated at OD 595 (Biotek).

### Synergy Calculation

Bliss and Loewe synergy scores were calculated in Python using the synergy package: https://pypi.org/project/synergy; source code https://github.com/djwooten/synergy(12).

### Animal Studies and Treatment

A total of 1 × 10^7^ MCF7 cells were resuspended in 50% Matrigel (BD) and injected into the third mammary fat pads of nulliparous NCR nude mice (Taconic) receiving 1 μmol/L β-Estradiol (Sigma) in drinking water, and palpable tumors were measured every 3 days. For the inhibitor studies, mice were treated daily once tumors exceeded 150 mm^3^. Alpelisib (MedChemExpress) was reconstituted in 0.5% methylcellulose (Sigma) and administered by oral gavage (35 mg/kg) once daily prior to tumor isolation and preparation. MI-503 (MedChemExpress) was reconstituted in 25% DMSO, 25% PEG400, and 50% PBS and administered by intraperitoneal injection (30 mg/kg) once daily prior to tumor isolation and preparation. Tumors were isolated and prepared within 3 hours of treatment. All mouse experiments were conducted in accordance with protocols approved by the Institutional Animal Care and Use Committee of Emory University School of Medicine.

### IHC

Formalin-fixed, paraffin-embedded prepared tumors were sectioned (Winship Cancer Tissue and Pathology Core) and mounted. Sections were deparaffinized, hydrated, and antigens retrieved with sodium citrate. Following blocking of endogenous peroxidases, sections were blocked and incubated in primary antibody overnight (4°C). Primary antibodies used were as follows: AKTS473 (Cell Signaling Technology 4060), H3K4me3 (Abcam 8580), and Ki67 (Abcam 16667). Sections were washed and incubated in secondary antibody, after which they were incubated with ABC (Vector Labs), DAB developed (Vector Labs), and counterstained with hematoxylin (Vector Labs). Sections were then dehydrated and mounted for imaging (Zeiss Observer A1) and images quantitated using Aperio ImageScope 12.1 (Leica).

### RNA Isolation, Sequencing, and Analysis

T47D cells were treated with 4 μmol/L MI-503 for 24 hours and RNA was isolated using the RNeasy Isolation Kit (Qiagen) according to the manufacturer's instruction. Preparation of RNA library and transcriptome sequencing was conducted by Novogene Co., LTD and transcript abundance was quantified using salmon (Illumina DRAGEN). Differentially expressed genes were identified using DESeq2 and genes with adjusted *P* value < 0.05 and |log2(FoldChange)| > 2 were considered differentially expressed. Gene set enrichment analysis (GSEA) of the preranked gene list was performed using clusterProfiler and fgsea, using the msigdb oncogenic signature gene set.

### CUT&RUN, Sequencing, and Analysis

T47D cells were treated with 4 μmol/L MI-503 for 24 hours after which cells were collected using accutase. CUT&RUN was performed using 200,000 input cells/condition with the CUT&RUN kit (Epicypher), according to the CUTANA CUT&RUN protocol v2.0 (Epicypher). Primary antibodies used were as follows: MLL1 (Epicypher 13-2004) and H3K4me3 (Abcam 8580). CUT&RUN-enriched DNA (5 ng) was used for subsequent library preparation according to manufacturer's instructions (Epicypher, CUTANA CUT&RUN Library Prep Kit). The resulting libraries were used for PE150 sequencing using the Novaseq 6000 platform at a depth of 5 GB/sample by Novogene Co., LTD. Adaptor trimming was performing using Trim Galore version 0.6.7. Sequences were then aligned to the human reference genome GRCh38.p14 using bowtie2 version 2.2.5. Equal amounts of fragmented Escherichia coli (E.coli) spike-in DNA were added to each sample to normalize read depth. Aligned sequences were sorted using samtools version 1.6, and duplicate reads were removed using the Picard Tools version 2.27.4. Heatmaps were generated using deeptools 3.5.1.

### Statistical Analysis

All experiments were performed in three independent experiments unless otherwise noted. Mean ± SEM are reported unless otherwise noted. Statistical significance (*P* < 0.05) of differences between two groups was determined by Student *t* test.

### Data Availability Statement

The data generated in this study are publicly available in the Gene Expression Omnibus at GSE214333.

## Results

### Combined PI3K/MLL1 Inhibition Reduces Breast Cancer Cell Line Clonogenicity Through On-target Activity

**FIGURE 1:**
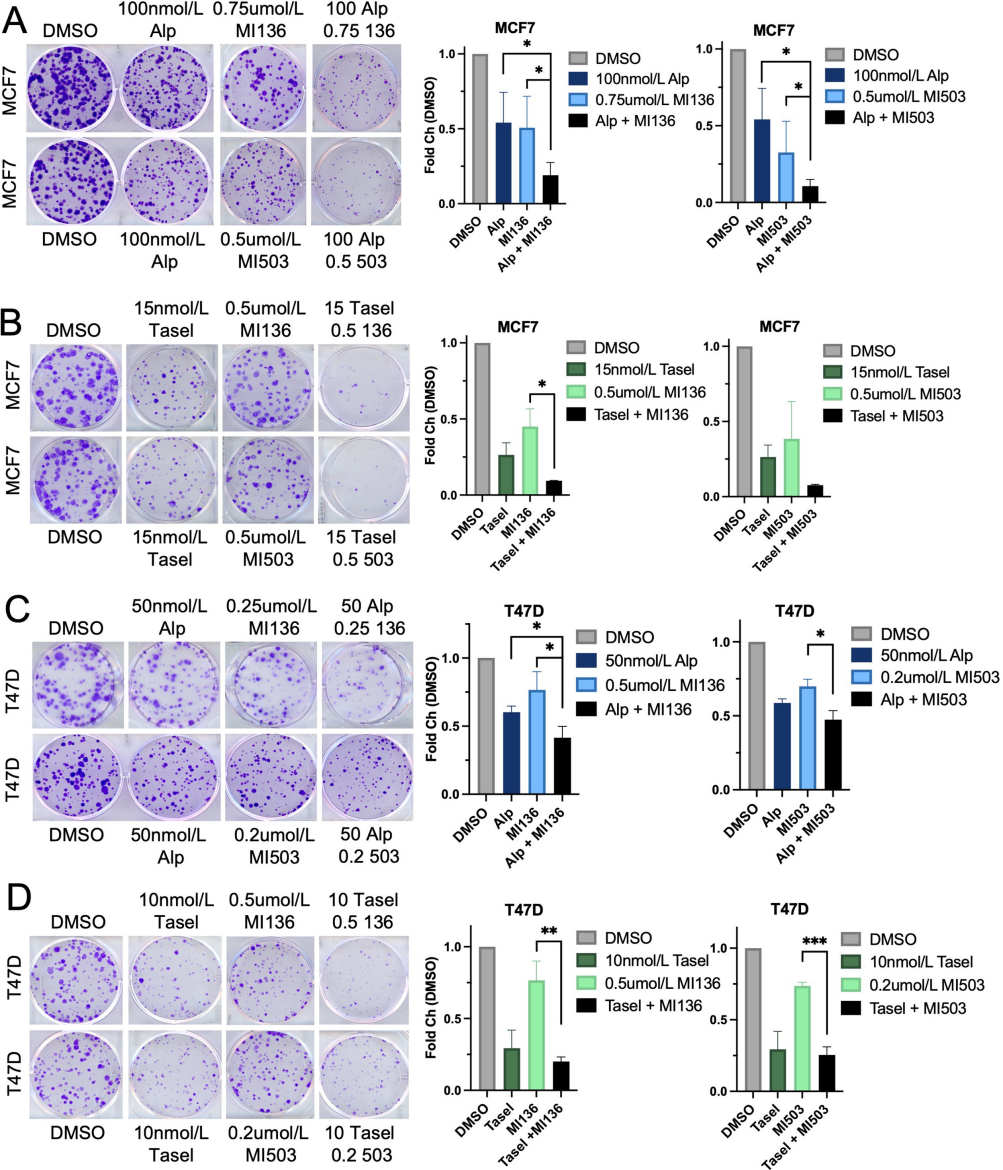
Combined PI3K and MLL1 inhibition reduces clonogenicity of HR^+^, PIK3CA-mutant, breast cancers. **A,** MCF7 breast cancer cells treated with the indicated concentrations of alpelisib, MI-136, MI-503, or DMSO for 19 days before fixation and crystal violet staining. **B,** MCF7 breast cancer cells treated with the indicated concentrations of taselisib, MI-136, MI-503, or DMSO for 19 days before fixation and crystal violet staining. **C,** T47D breast cancer cells treated with the indicated concentrations of alpelisib, MI-136, MI-503, or DMSO for 19 days before fixation and crystal violet staining. **D,** T47D breast cancer cells treated with the indicated concentrations of taselisib, MI-136, MI-503, or DMSO for 19 days before fixation and crystal violet staining. For all experiments, representative images from at least three independent experiments are shown. Data are shown as mean ± SEM. *, *P* < 0.05; **, *P* < 0.01; ***, *P* < 0.001.

Aberrant PI3K/AKT signaling regulates H3K4 methylation through the activity of H3K4-directed enzymes in HR^+^ breast cancer (6, 7) and high H3K4 methylation is associated with poor prognosis in breast and other cancers (8). We postulated that combined PI3K and MLL inhibition may provide additional therapeutic benefit in this context. To assess the effects of combined PI3K/MLL1 inhibition on cell growth, the PI3K-activated (PIK3CA^E545K^-mutant), HR^+^ breast cancer cell line MCF7 was treated with the p110α-selective PI3K inhibitor alpelisib, the MLL1 inhibitor MI-503, or the combination. Combined PI3K and MLL1 inhibition reduced the clonogenicity and cell proliferation compared with either monotherapy (Fig. 1A; Supplementary Fig. S1A). MI-503 is a small-molecule MLL1 inhibitor that disrupts the association between MLL1 and the MLL1 COMPASS complex cofactor Menin, thereby reducing promoter H3K4me3 and subsequent target gene expresion (13). Cells treated with the MLL-Menin inhibitor MI-136 (14) in combination with PI3K inhibitors also exhibit impaired clonogenicity (Fig. 1A). Similar results were observed in cells treated with MLL1 inhibitors in combination with the p110β-sparing PI3K inhibitor taselisib (ref. 15; Fig. 1B) or the pan-PI3K inhibitor pictilisib (ref. 16; Supplementary Fig. S1B). Combined PI3K and MLL1 inhibition also decreased clonogenicity in the HR^+^, PIK3CA^H1047R^-mutant cell line T47D (Fig. 1C and D; Supplementary Fig. S1C). These data suggest that combined PI3K and MLL1 inhibition impairs the clonogenicity in PI3K-activated, HR^+^, breast cancer models.

**FIGURE 2:**
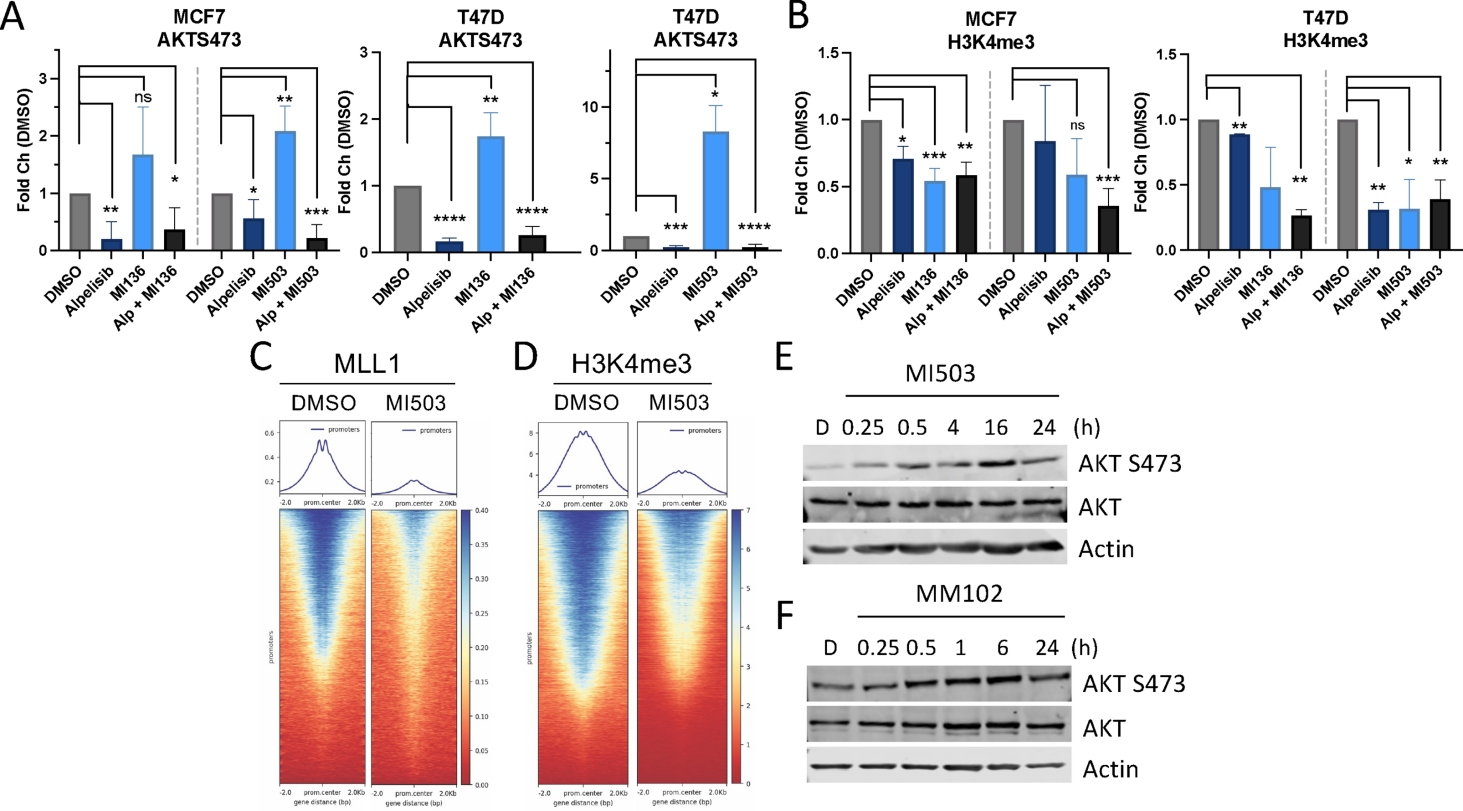
MLL1 inhibition suppresses H3K4 methylation in HR^+^, PIK3CA-mutant, breast cancers. **A,** Protein quantification for MCF7 or T47D breast cancer cells treated with alpelisib (1 μmol/L), MI-136 (4 μmol/L), MI-503 (4 μmol/L), or DMSO for 24 hours prior to protein extraction. Lysates were immunoblotted for the indicated antibodies. **B,** Protein quantification for MCF7 or T47D breast cancer cells treated with alpelisib (1 μmol/L), MI-136 (4 μmol/L), MI-503 (4 μmol/L), or DMSO for 96 hours prior to acid extraction of histones. Lysates were immunoblotted for the indicated antibodies. Gene metanalysis and promoter heatmap centered ±2 kb around transcriptional start sites in T47D cells treated with DMSO or 4 μmol/L MI-503 for 24 hours prior to MLL1 (**C**) and H3K4me3 (**D**) CUT&RUN. *n* = 2 independent experiments, representative plot shown. **E,** T47D cells treated with 4 μmol/L MI-503 for the indicated times and then lysates prepared. Lysates were immunoblotted for the indicated antibodies. **F,** T47D cells treated with MM102 (6 μmol/L) for the indicated time and then lysates prepared. Lysates were immunoblotted for the indicated antibodies.

We next determined whether the biological activity of PI3K and MLL1 inhibitors observed is a result of on-target enzymatic inhibition. MCF7 and T47D cells were treated with PI3K and/or MLL1 inhibitors, and PI3K inhibition with alpelisib reduced effector signaling as indicated by a decrease in phosphorylated AKT (Fig. 2A; Supplementary Fig. S2A) and MLL inhibition modestly reduced H3K4 methylation (Fig. 2B; Supplementary Fig. S2B). Similar results were observed with the PI3K inhibitors taselisib and pictilisib (Supplementary Fig. S2C and S2D). Combined PI3K and MLL1 inhibition reduced H3K4 methylation and was detectable as early as 96 hours (Supplementary Fig. S2E). These results are similar to MLL1-inhibitor monotherapy-directed loss of H3K4 methylation, which was also detected at later timepoints such as 96 hours (Fig. 2B) and is consistent with the time required to detect global changes to histone PTMs in response to chemical inhibition (6). Both MLL1 genomic promoter occupancy (Fig. 2C) and promoter-associated H3K4me3 (Fig. 2D) is reduced following MLL1 inhibition for 24 hours, suggesting on-target activity of the MLL1 inhibitor MI-503. These data support the hypothesis that the reduction of H3K4me3 associated with MLL1 inhibition is a result of a loss of MLL1 binding and subsequent methylation.

### MLL Inhibition Induces Feedback Activation of AKT Signaling

Unexpectedly, MLL inhibition in PI3K-activated cancer cell line models increased PI3K effector signaling as indicated by increased AKT phosphorylation, which was abrogated with combined PI3K and MLL inhibition (Fig. 2A; Supplementary Fig. S2A–S2C). The detected increase in effector signaling following MLL1 monotherapy suggests the presence of a feedback loop between MLL and AKT. The MLL1 inhibitor–mediated increase in AKT phosphorylation was detected as early as 15 minutes, and was sustained for at least 24 hours (Fig. 2F). To test whether the ability of MLL1 inhibition to enhance PI3K effector activity is due to on-target inhibitor activity, we treated T47D cells with MLL1 inhibitors that function through disparate mechanisms of action and found that disruption of MLL1 interaction with the MLL1/COMPASS cofactor WDR5 through the peptidomimetic compound MM102 (17) increased AKT phosphorylation (Fig. 2G). These data suggest a functional relationship between PI3K/AKT signaling and MLL methyltransferases which may support their combined inhibition.

**FIGURE 3:**
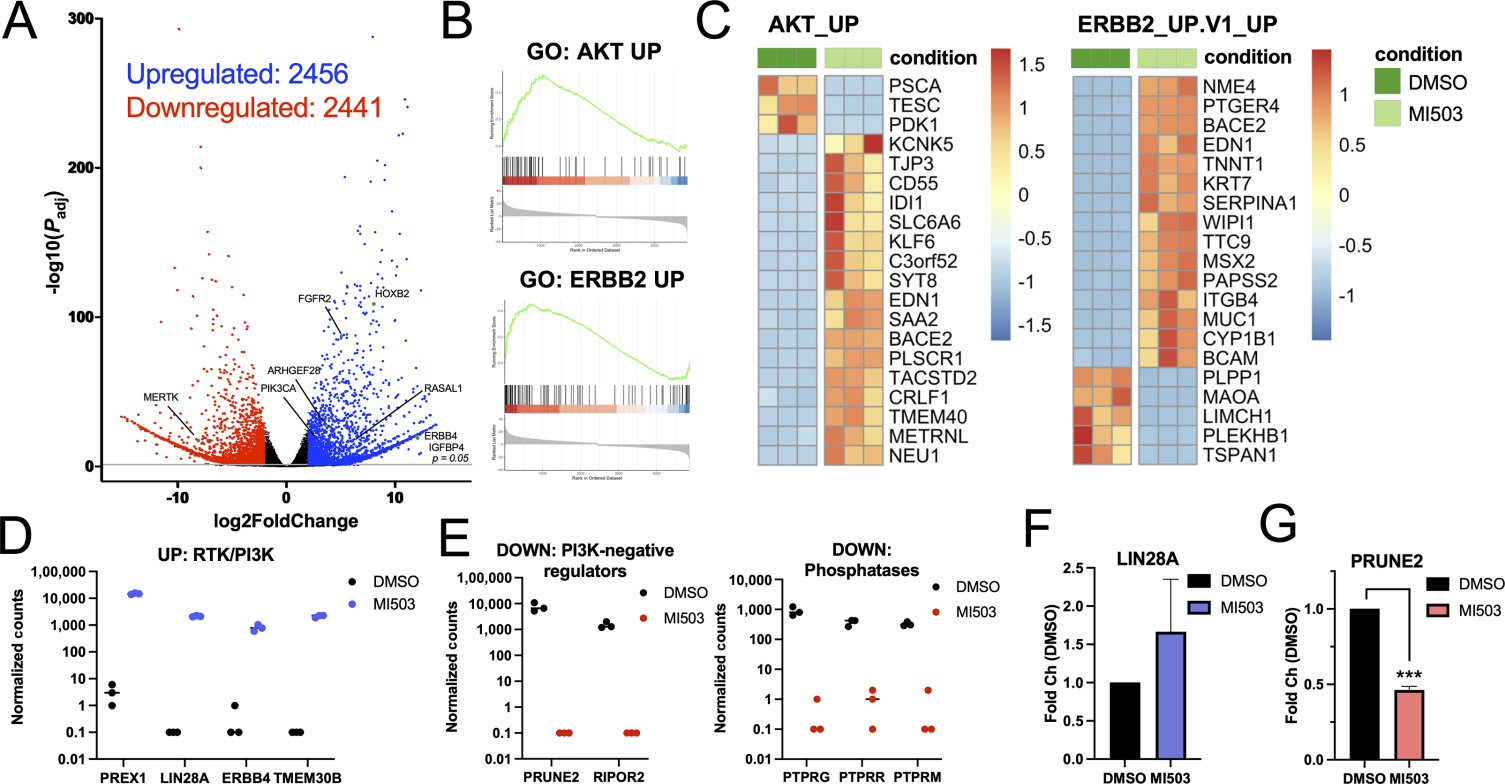
MLL1 inhibition hyperactivates AKT through a feedback loop. **A,** Volcano plot of statistical significance (*P* = 0.05) against fold change between T47D cells treated with control (DMSO) and MI-503 (4 μmol/L) for 24 hours. **B,** GSEA depicting GO classifications of differentially expressed gene sets between the DMSO and MI-503 cohorts shown in **A**. **C,** Heatmaps representing the top 20 differentially expressed genes from gene ontology (GO) classifications shown in **B**. Normalized expression of PI3K-activating, upregulated genes (**D**) and normalized expression of PI3K-suppresive, downregulated genes (**E**) from *n* = 3 independent experiments (**A**). T47D cells treated with control (DMSO) or 4 μmol/L MI-503 for 24 hours. Lysates were immunoblotted for the indicated antibodies. *n* = 2 (**F**) and 3 (**G**) independent experiments.

To provide mechanistic insight into how MLL inhibition remodels chromatin to alter gene expression to support AKT activation, we performed RNA sequencing (RNA-seq) on T47D cells treated with the MLL inhibitor MI-503 for 24 hours. Expression of more than 2,400 genes was upregulated following MLL inhibition, including genes involved in upstream receptor tyrosine kinase signaling (*FGFR2*, *ERBB4*, *IGFBP4*), and PI3K/RAS signaling (*PIK3CA*; Fig. 3A). More than 2,400 downregulated genes were also identified in the MLL1 inhibitor treatment group (Fig. 3A), which is consistent with the reduction of promoter H3K4me3 and subsequent downregulation of gene expression functioning as the predominant mechanism of action for MLL inhibitors (18). MLL inhibition significantly dysregulated the expression of biological pathways and processes, including upregulation of ERBB2, RAS, RAF, mTOR, and AKT, which promote AKT and AKT effector activation (Supplementary Fig. S3A). GSEA further demonstrated that MLL inhibition significantly upregulates the expression of AKT and ERBB2 pathway genes (Fig. 3B and C; Supplementary Fig. S3B). RNA-seq specifically detected increased expression of genes that positively regulate PI3K/AKT signaling, including phospholipid transport proteins (*TMEM30b*), receptor tyrosine kinases (*ERBB4*), and an RNA-binding protein that activates PI3K signaling via blocking expression of the tumor suppressor miRNA let7 (*LIN28A*; Fig. 3D). Alternatively, MLL1 inhibition downregulates PI3K negative regulators including tyrosine phosphatases, and suppressors of RAS signaling (*PRUNE2*, *RIPOR2*; Fig. 3E). MLL1 inhibition further increases LIN28A protein detection (Fig. 3F) and decreases PRUNE2 protein levels (Fig. 3G). MLL1 inhibition reduces both H3K4me3 and MLL1 association with the *PRUNE2* promoter (Supplementary Fig. S3C), suggesting that loss of PRUNE2 expression may contribute to the observed increase in AKT phosphorylation following MLL1 inhibition. Collectively, these data demonstrate MLL inhibition promotes the activation of AKT signaling possibly through the presence of a feedback loop downstream of MLL and upstream of AKT.

### Combined PI3K/MLL1 Inhibition Synergizes to Reduce Viability by Enhancing Apoptosis *In Vitro*

**FIGURE 4:**
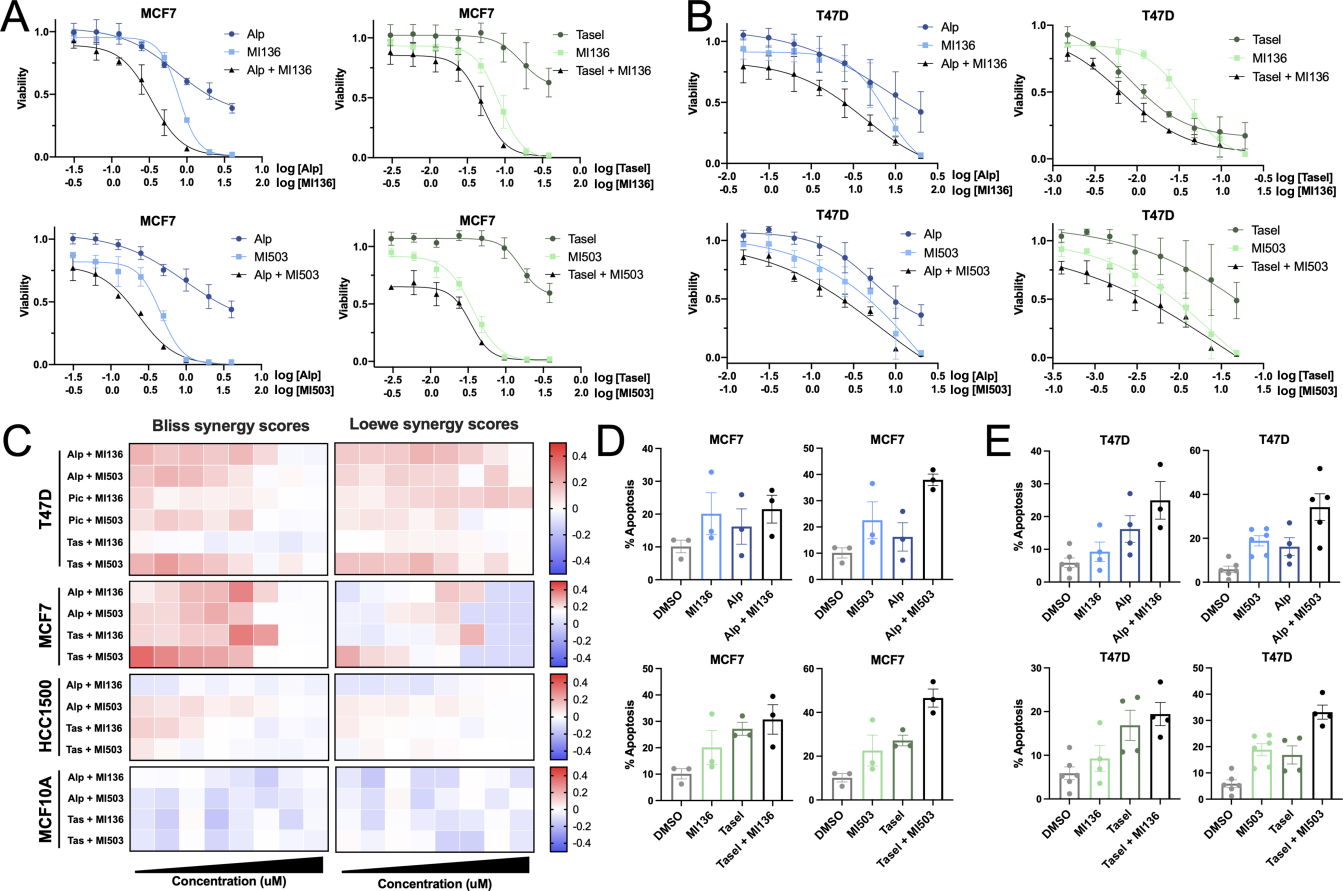
PI3K and MLL1 combination therapy synergizes to reduce viability and enhance apoptosis. **A,** Cell viability curves in MCF7 breast cancer cells treated with an 8-point range of DMSO, alpelisib, taselisib, MI-503, and/or MI-136 for 120 hours. Results shown are representative of at least three independent experiments. Data are shown as mean ± SEM. **B,** Cell viability curves in T47D breast cancer cells treated with an 8-point range of DMSO, alpelisib, taselisib, MI-503, and/or MI-136 for 120 hours. Results shown are representative of at least three independent experiments. Data are shown as mean ± SEM. **C,** Bliss (left) and Loewe (right) synergy calculated from MCF7, T47D, HCC1500, and MCF10A datapoints in **A**, **B**, and Supplementary Fig. S4. **D,** Annexin V staining in MCF7 cells treated with DMSO, alpelisib (1 μmol/L), taselisib (0.5 μmol/L), MI-136 (4 μmol/L), MI-503 (4 μmol/L) for 120 hours. Results shown are representative of at least three independent experiments. Data are shown as mean ± SEM. **E,** Annexin V staining in MCF7 cells treated with DMSO, alpelisib (1 μmol/L), taselisib (0.5 μmol/L), MI-136 (4 μmol/L), MI-503 (4 μmol/L) for 120 hours. Results shown are representative of at least three independent experiments. Data are shown as mean ± SEM.

A major limitation of clinical PI3K inhibition is the lack of apoptotic induction that leads to tumor clearance, as PI3K monotherapy is primarily associated with a cytostatic effect induced by G_1_ arrest (19). To determine whether combined PI3K/MLL1 inhibition overcomes PI3K inhibitor–induced cytostasis, we treated PI3K-activated, HR^+^ breast cancer cells with the PI3K inhibitor alpelisib, the MLL1 inhibitor MI-503, or the combination, and evaluated cellular viability. Combined PI3K and MLL inhibition reduced cell viability in multiple PI3K-activated breast cancer models (Fig. 4A and B), which was confirmed with additional PI3K and MLL1 inhibitors (Supplementary Fig. S4A). Dual PI3K/MLL inhibition selectively reduced the viability of PIK3CA-mutated, HR^+^ breast cancer cell lines, as the viability of both the PI3K pathway wildtype, HR^+^ breast cancer cell line HCC1500 and the normal breast cell line MCF10A remained unchanged (Supplementary Fig. S4B and S4C). We next aimed to determine whether PI3K and MLL1 combination therapy is synergistic, as synergistic regimens potentially maximize therapeutic effects while minimizing off-target effects (20). Drug synergy was calculated using both Loewe and Bliss, which measure synergy within the same pathway or among distinct pathways, respectively. Bliss and Loewe algorithms separately defined the combination of low-dose PI3K and MLL1 inhibition as synergistic; synergy was not achieved for the normal breast cell line MCF10A or the PI3K wildtype, HR^+^ breast cancer cell line HCC1500 (Fig. 4C; ref. 21). While PI3K or MLL1 monotherapies do not yield apoptotic induction, the combination of PI3K and MLL1 inhibition increased apoptosis (Fig. 4D and E), which is consistent with cell viability (Fig. 4A and B). To assess whether the detected loss of viability is due to on-target effects of MLL1 inhibition, we utilized the MLL1 peptiomimetic inhibitor MM102. Combined PI3K inhibition with MM102 reduces cell viability and proliferation in models of PI3K-activated breast cancers (Supplementary Fig. S4A). These data demonstrate that combined PI3K and MLL inhibition synergize to reduce viability in models of PI3K-activated, HR^+^, breast cancer.

### Dual PI3K/MLL1 Inhibition Reduces Tumor Growth in Xenograft Models of PIK3CA-activated, HR^+^ Breast Cancer

**FIGURE 5:**
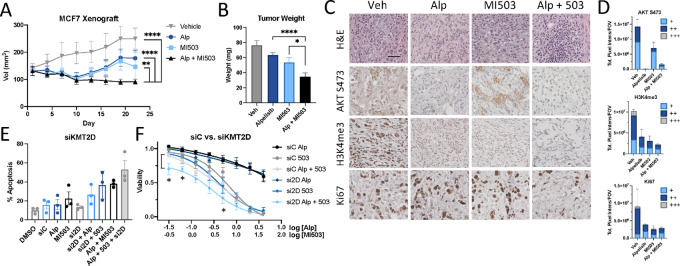
Combined MLL and PI3K inhibition provides therapeutic benefit in *in vivo* models of breast cancer. **A,** Tumor volume of MCF7 cells implanted into the mammary fat pads of nude mice with once-daily treatment with alpelisib (35 mg/kg, gavage), MI-503 (30 mg/kg, i.p.), or the combination. Endpoint tumor volume: MCF7 vehicle, 250.17 ± 11.24 mm^3^; MCF7 alpelisib, 178.11 ± 7.87 mm^3^; MCF7 MI-503, 146.59 ± 15.59 mm^3^; and MCF7 alpelisib/MI-503, 91.83 ± 2.69 mm^3^. Means ± SEM are shown; all groups *n* ≥ 12; two-way ANOVA with Tukey multiple comparisons test. **, *P* < 0.01; ****, *P* < 0.0001. **B,** The wet weight of individual mammary tumors plus associated mammary gland tissue from MCF7 vehicle (*n* = 12), MCF7 alpelisib (*n* = 13), MCF7 MI-503 (*n* = 11), and MCF7 alpelisib/MI-503 (*n* = 12). Tumors were harvested 22 days from treatment onset. Means ± SEM are shown. *, *P* < 0.05; ****, *P* < 0.0001; unpaired *t* test. **C,** Tumors derived from mice in **A**. Sections are stained with the indicated antibodies, randomly selected images shown. (Scale bar, 40 μm.) *n* = 3 mice/condition. **D,** Quantification of IHC. *n* = 3 mice/condition, 2 fields of view (FOV)/mouse. Total pixel/FOV quantitated as low (+), medium (++), or high (+++). Statistical analyses shown in Supplementary Table S1. **E,** Annexin V staining in MCF7 cells transfected with MLL4/KMT2D or control siRNA and treated with DMSO, alpelisib (1 μmol/L), and/or MI-503 (4 μmol/L) for 120 hours. *n =* 3 independent experiments. Data are shown as mean ± SEM. **F,** Cell viability curve in MCF7 breast cancer cells reverse transfected with siControl or siKMT2D to genetically inhibit KMT2D/MLL4 and treated with an 8-point range of DMSO, alpelisib and/or MI-503 for 120 hours. *n =* 4 independent experiments. Data are shown as mean ± SEM.

To determine whether HR^+^ breast tumors characterized by PI3K pathway activation are sensitive to dual PI3K/MLL1 inhibition, MCF7 cells were transplanted into the mammary fat pads of nude mice. Palpable tumors were treated with vehicle, alpelisib, MI-503, or the combination. While alpelisib-treated and MI-503–treated tumors were smaller than vehicle-treated tumors, combined PI3K and MLL1 inhibition significantly reduced tumor volume (Fig. 5A) and endpoint tumor weight (Fig. 5B), without toxicity-associated changes to animal weight during the treatment duration (Supplementary Fig. S5A). Dual PI3K and MLL1 inhibition reduced PI3K effector phosphorylation, H3K4 methylation, and cellular proliferation (Fig. 5C and D; Supplementary Fig. S5B), which is quantified in Supplementary Table S1. MLL1 inhibition via MI-503 monotherapy retained AKT phosphorylation (Fig. 5C and D), which was consistent with *in vitro* MLL1 inhibition (Fig. 2; Supplementary Fig. S2). Taken together, our preclinical data suggest that HR^+^ breast xenografts characterized by PIK3CA mutation exhibit therapeutic benefit from combined PI3K and MLL1 inhibition that exceeds PI3K or MI-503 monotherapy. Furthermore, patients with this subset of breast tumors may be suitable candidates for dual PI3K and MLL1 inhibition.

### MLL4/KMT2D Genetic Ablation Enhances PI3K/MLL1 Inhibitor–driven Apoptosis and Reduction in Proliferation

Most MLL inhibitors in preclinical and clinical investigation specifically target the MLL1 COMPASS complex (13, 14, 17). Because H3K4 methylation is elevated in breast and other cancers and is associated with a poor patient prognosis (8), MLL1 inhibition may function as a viable therapeutic target. However, published reports suggest that the MLL enzyme MLL4/KMT2D is an AKT substrate, and its enzymatic activity is associated with resistance to the PI3K inhibitor alpelisib in HR^+^ breast cancer via epigenome-driven upregulation of ER signaling (7). To address whether the loss of KMT2D enhances the observed synergy with dual PI3K and MLL1 inhibition, we utilized siRNA to knock down KMT2D (Supplementary Fig. S5C). Combined PI3K/MLL1 inhibition with KMT2D knockdown increased apoptosis compared with all single- and double-agent treatment groups (Fig. 5E; Supplementary Fig. S5D–S5F) in the MCF7 cell line. Dual PI3K and MLL1 inhibition in combination with KMT2D knockdown further reduced cell viability at low inhibitor doses (Fig. 5F). These data suggest that enzymatic inhibition of MLL1 and KMT2D/MLL4, when combined with PI3K inhibition, is a promising therapeutic modality to induce cell death and produce a durable therapeutic response in PI3K-activated cancers.

## Discussion

Of the approximately 70% of breast cancers that are HR^+^, 40% are characterized by aberrant PI3K pathway activation. The critical role of PI3K signaling in carcinogenesis has prompted the development and clinical testing of pan- and isoform-selective PI3K inhibitors in this patient population. However, the utility of PI3K-targeted therapies is limited as PI3K inhibitors generally lack efficacy as monotherapies. Thus, research to define novel mechanisms by which PI3K/AKT contributes to oncogenesis and exploiting these mechanisms to develop and test combination therapies for the treatment of PI3K-activated cancers is critical. Here, we build upon previous studies demonstrating that the PI3K effector AKT regulates cellular H3K4 methylation through the phosphorylation of the H3K4 methyltransferase KMT2D/MLL4 (7) and the H3K4 demethylase KDM5A (6) in breast cancer. AKT-mediated KDM5A phosphorylation redistributes KDM5A from the nucleus into the cytoplasm, rendering it ineffective at H3K4me2/3 demethylation (6). In this context, elevated promoter H3K4me3 supports the transcription and expression of cell cycle–promoting genes, encouraging proliferation and oncogenesis. In contrast, AKT- or SGK2-driven phosphorylation of the H3K4 methyltransferase KMT2D/MLL4 attenuates its activity to reduce enhancer H3K4 methylation (7, 22). PI3K inhibition enhances KMT2D activity, further driving H3K4 methylation and promoting an open chromatin conformation, primarily at enhancers typically bound by the FOXA1 pioneer factor that also contain ER binding motifs. The open chromatin conformation supports FOXA1 binding, which then recruits ERα to initiate the transcription of ER-regulated genes. These disparate mechanisms of action drive biologically distinct and separable H3K4 methylation events under conditions of either PI3K pathway hyperactivation or inhibition, suggesting that both promoter and enhancer H3K4 methylation is downstream of the PI3K/AKT pathway, and that combined PI3K and MLL inhibition may provide therapeutic benefit.

Cytostatic activity represents a significant limitation of clinical PI3K inhibition (19). Here we demonstrate that the combined inhibition of p110α and MLL1 synergizes to induce apoptosis of PIK3CA-mutant, HR^+^, breast cancer cell lines. Dual PI3K and MLL1 inhibition reduces the clonogenicity of PIK3CA-mutant breast cancer cell lines, while also decreasing their proliferation. This synergy is enhanced by genetic ablation of KMT2D/MLL4. Furthermore, we identify a compensatory feedback loop between MLL methyltransferases and the PI3K pathway. MLL1 inhibitor–driven feedback AKT activation correlates with both reduced expression of negative PI3K pathway regulators and enhanced expression of upstream pathways and proteins that can contribute to AKT activation. Because MLL1 inhibition primarily reduces promoter H3K4me3 to repress gene expression (Figs. 2 and 3), the mechanism(s) that may lead to enhanced gene expression following MLL1 inhibition are not well characterized. While the possibility of MLL-inhibitor off-target effects cannot be eliminated, AKT hyperactivation following the use of divergent MLL inhibitors suggests that compensatory AKT activation is the result of on-target MLL inhibition. Functionally, MLL1 inhibition may result in the compensatory activation of other MLL COMPASS complexes (e.g., MLL2), that could trigger an increase in methylation and subsequent gene expression at select genomic loci. MLL2 compensation has been described in other contexts, including MLL1 deletion in MLL1-AF9 translocated leukemias (23). MLL1 function has also been shown to be redundant with MLL2 at bivalent promoters in murine embryonic stem cells (24). Collectively, these scenarios may partially explain why we observe a dramatic reduction in MLL1 promoter localization without an equivalent reduction of promoter H3K4me3 (Fig. 2).

Here we also define repressed gene transcripts who function(s) may support the MLL1 inhibitor–directed feedback activation of AKT. Our results support a hypothesis in which MLL1 inhibition reduces MLL1 genomic occupancy at promoters to reduce promoter H3K4me3, thereby repressing gene expression. MLL1 inhibition reduces the expression of more than 2,000 genes and includes PI3K/RAS/AKT negative regulators such as PRUNE2. PRUNE2 is a tumor suppressor in prostate and other cancers, inhibiting Ras homolog family member A (RhoA) activity to support oncogenic transformation (25). We found that MLL1 inhibition reduces *PRUNE2* transcript expression and protein detection, supporting a role of PRUNE2 dysregulation in MLL1 inhibitor–associated AKT activation. While additional experiments are required to fully dissect the mechanism(s) that underlie the observed MLL feedback loop, AKT hyperactivation occurs relatively quickly and is conserved among MLL inhibitors that exhibit divergent scaffolding and thus function through different mechanisms—through MLL1/WDR interaction, or via MLL1/Menin binding. The placement of MLL both upstream and downstream of AKT in the PI3K signaling network highlights the importance of dual inhibition of these enzymes as a mechanism to durably suppress PI3K pathway activation.

Our findings suggest that while PI3K and MLL1 inhibition is an effective therapeutic strategy in PIK3CA-mutant, HR^+^ breast cancer cell lines, this combination can be improved. Current MLL1 inhibitors specifically target MLL1 through protein–protein interactions with essential MLL1-specific COMPASS members WDR5 or Menin. MLL1 methyltransferase activity is dependent on its association with WDR5; high-affinity peptidomimetic inhibitors including MM102 bind WDR5 to prevent association with MLL1 (17). Other MLL1 inhibitors including MI-136 and MI-503 share a thienopyrimidine scaffolding and function via abrogation of the MLL1/Menin association to induce cell death in models of MLL1-translocated leukemias and androgen receptor–dependent prostate cancer (14, 26). While we demonstrate that these MLL1 inhibitors synergize with PI3K inhibitors in PIK3CA-mutant breast cancer, this combination does not effectively target the MLL4/KMT2D COMPASS complex, which is a functionally distinct downstream PI3K effector that is essential in mediating resistance to endocrine therapies including fulvestrant (7). Because KMT2D/MLL4 or pan-MLL inhibitors that target the enzymatic activity of the SET-domain have only recently been published (27) and are not commercially available, we genetically ablated KMT2D in combination with PI3K/MLL1 dual inhibition and found that knockdown of KMT2D further enhances the PI3K/MLL1-driven apoptosis (Fig. 4). The results presented here suggest a therapeutic opportunity to design and test KMT2D/MLL4 or pan-MLL inhibitors in PI3K-activated, HR^+^ breast cancers.

## Supplementary Material

Figure S1 Figure S1 shows combined effects of PI3K and MLL1 inhibition in clonogenic assays

Figure S2 Figure S2 shows on-target activity of PI3K and MLL1 inhibitors

Figure S3 Figure S3 shows additional transcriptional and genomics analyses that support MLL1 inhibitor-driven hyperactivation of AKT

Figure S4 Figure S4 shows combined PI3K and MLL1 inhibition reduces cell viability and enhances apoptosis

Figure S5 Figure S5 shows that combined PI3K and MLL inhibition provides therapeutic benefit in vitro and in vivo

Supplementary Table ST1 Supplementary Table 1 shows p values for IHC staining that corresponds to the graphs shown in Figure 5D
