# Supplementary material for: Targeting MLL Methyltransferases Enhances the Antitumor Effects of PI3K Inhibition in Hormone Receptor–positive Breast Cancer
Source: Cancer Res Commun. 2022 Dec 6;2(12):1569–78. doi: 10.1158/2767-9764.CRC-22-0158 (PMC10036132; doi:10.1158/2767-9764.CRC-22-0158)
Supplement: Figure S1 — shows combined effects of PI3K and MLL1 inhibition in clonogenic assays [file crc-22-0158-s01.docx]

**Supplementary** **Figure 1. Combined PI3K and MLL1 inhibition reduces clonogenicity of HR+, PIK3CA-mutant, breast cancers**. (A) MCF7 breast cancer cells treated with the indicated concentrations of alpelisib, MI-136, or DMSO for 17 days before fixation and crystal violet staining. Representative images shown. (B) MCF7 breast cancer cells treated with the indicated concentrations of pictilisib, MI-136, MI-503, or DMSO for 17 days before fixation and crystal violet staining. Representative images shown. Results shown are representative of at least 3 independent experiments. Data are shown as mean ± SEM. **P* < 0.05, unpaired t-test. (C) T47D breast cancer cells treated with the indicated concentrations of pictilisib, MI-136, or DMSO for 19 days before fixation and crystal violet staining. Representative images shown. Results shown are representative of at least 3 independent experiments. Data are shown as mean ± SEM.
