# Supplementary material for: Targeting MLL Methyltransferases Enhances the Antitumor Effects of PI3K Inhibition in Hormone Receptor–positive Breast Cancer
Source: Cancer Res Commun. 2022 Dec 6;2(12):1569–78. doi: 10.1158/2767-9764.CRC-22-0158 (PMC10036132; doi:10.1158/2767-9764.CRC-22-0158)
Supplement: Figure S2 — shows on-target activity of PI3K and MLL1 inhibitors [file crc-22-0158-s02.docx]

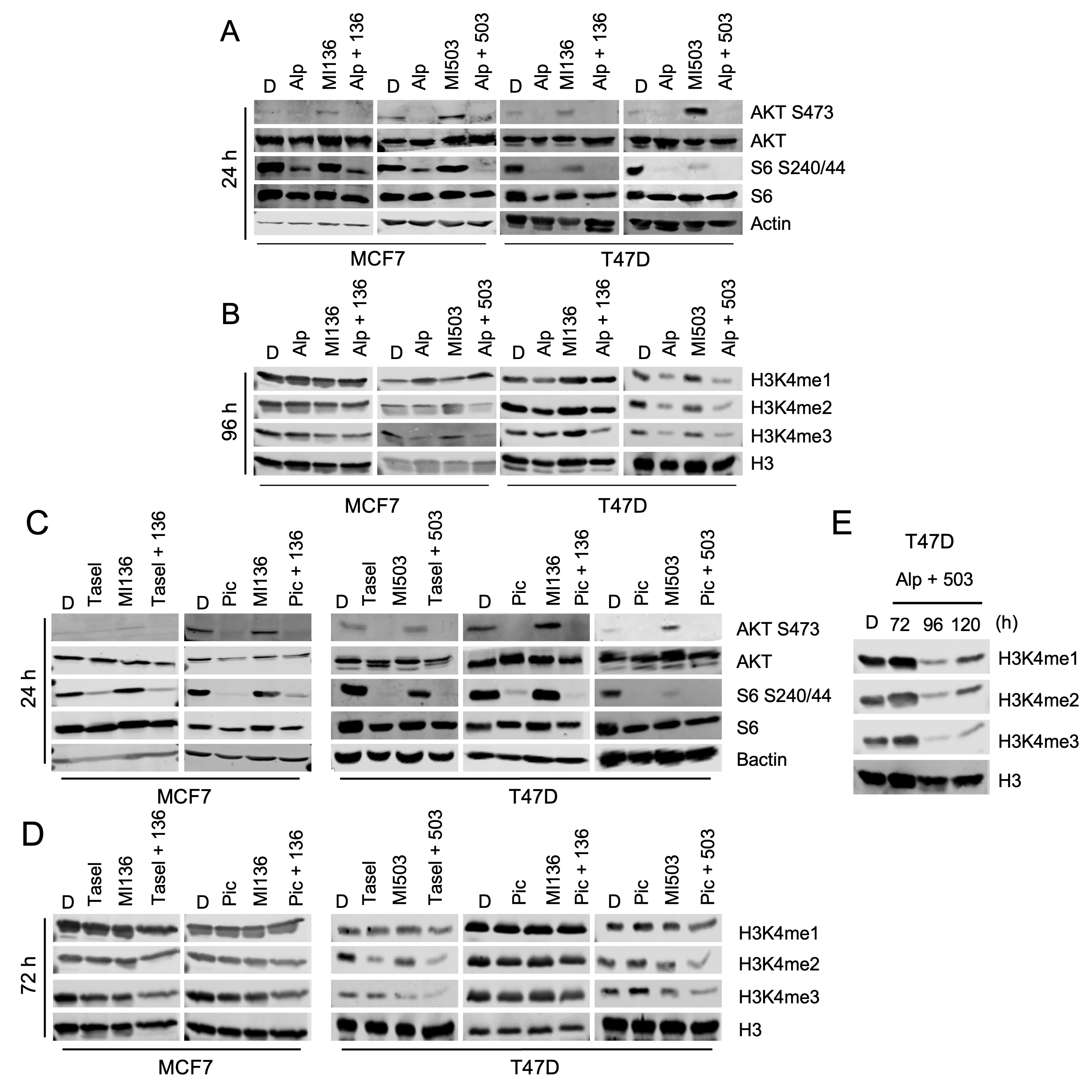


**Supplementary** **Figure 2: MLL1 inhibition hyperactivates AKT**. (A) MCF7 (left) or T47D (right) breast cancer cells were treated with alpelisib (1 uM), MI-136 (4 uM), MI-503 (4 uM) or DMSO for 24h prior to protein extraction. Lysates were immunoblotted for the indicated antibodies. (B) MCF7 (left) or T47D (right) were treated with alpelisib (1 uM), MI-136 (4 uM), MI-503 (4 uM) or DMSO for 96h prior to acid extraction. Lysates were immunoblotted for the indicated antibodies. (C) MCF7 (left) or T47D (right) were treated with taselisib (1 uM), pictilisib (1 uM), MI-136 (4 uM), MI-503 (4 uM) or DMSO for 24h prior to protein extraction. Lysates were immunoblotted for the indicated antibodies. (D) MCF7 (left) or T47D (right) were treated with taselisib (1 uM), pictilisib (1 uM), MI-136 (4 uM), MI-503 (4 uM) or DMSO for 72h prior to acid extraction. Lysates were immunoblotted for the indicated antibodies. (E) T47D cells treated with alpelisib (1 uM) or MI503 (4 uM) for the indicated times and lysates acid extracted and immunoblotted for the indicated antibodies.
