# Supplementary material for: Targeting MLL Methyltransferases Enhances the Antitumor Effects of PI3K Inhibition in Hormone Receptor–positive Breast Cancer
Source: Cancer Res Commun. 2022 Dec 6;2(12):1569–78. doi: 10.1158/2767-9764.CRC-22-0158 (PMC10036132; doi:10.1158/2767-9764.CRC-22-0158)
Supplement: Figure S3 — shows additional transcriptional and genomics analyses that support MLL1 inhibitor-driven hyperactivation of AKT [file crc-22-0158-s03.docx]

**Supplementary** **Figure 3: MLL1 inhibition hyperactivates PI3K effector signaling via induction of gene expression profile changes**. (A). Table of enrichment graphs depicting the top 10 pathways enriched at the top and bottom of the ranked gene list, generated using the oncogenic signature gene set (c6.all.v7.4.symbols.gmt). (B) Stripplots showing differences in the normalized enrichment scores (TPM) of the top 20 genes identified as significantly enriched in the GSEA analysis between control (DMSO) and MI503. (C) PRUNE2 H3K4me3 and MLL1 IGV tracks.
