# Supplementary material for: Targeting MLL Methyltransferases Enhances the Antitumor Effects of PI3K Inhibition in Hormone Receptor–positive Breast Cancer
Source: Cancer Res Commun. 2022 Dec 6;2(12):1569–78. doi: 10.1158/2767-9764.CRC-22-0158 (PMC10036132; doi:10.1158/2767-9764.CRC-22-0158)
Supplement: Figure S4 — shows combined PI3K and MLL1 inhibition reduces cell viability and enhances apoptosis [file crc-22-0158-s04.docx]

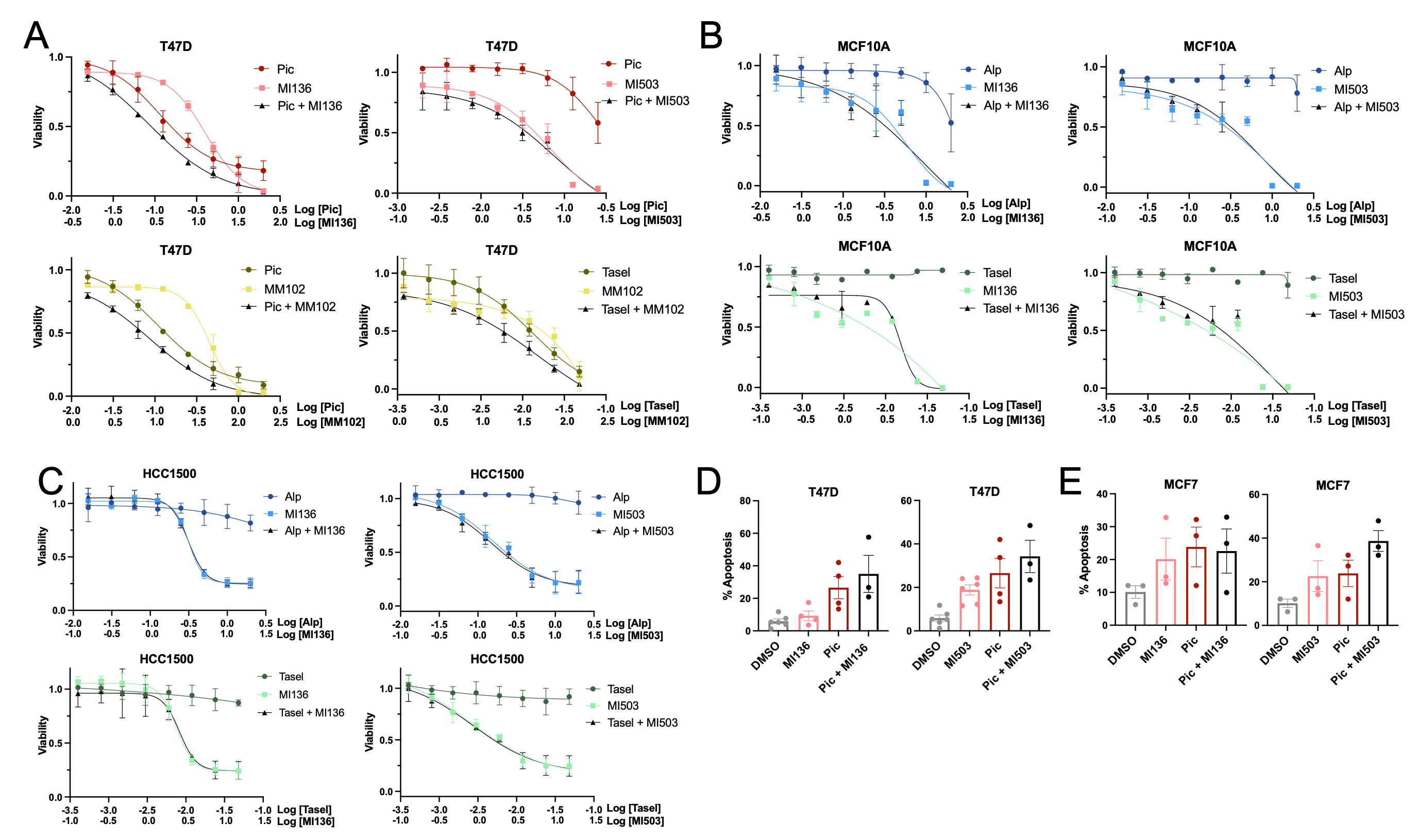


**Supplementary** **Figure 4: PI3K and MLL1 combination therapy synergizes to reduce viability and enhance apoptosis.** (A) Cell viability curves in T47D breast cancer cells treated with an 8-point range of DMSO, pictilisib, taselisib, MI-503, MI-136 and/or MM-102 for 120h. *n* ≥ at least 3 independent experiments. Data are shown as mean ± SEM. (B) Cell viability curves in MCF10A normal breast cells treated with an 8-point range of DMSO, alpelisib, taselisib, MI-136, and/or MI-503 for 120h. *n* ≥ 3 independent experiments. Data are shown as mean ± SEM. (C) Cell viability curves in HCC1500 breast cancer cells treated with an 8-point range of DMSO, alpelisib, taselisib, MI-136, and/or MI-503 for 120h. *n* ≥ 3 independent experiments. Data are shown as mean ± SEM. (D) Annexin V staining in T47D cells treated with DMSO, pictilisib (1 uM), MI-136 (4 uM), MI-503 (4 uM) for 120h. Data are shown as mean ± SEM; n = 3. (E) Annexin V staining in MCF7 cells treated with DMSO, pictilisib (1 uM), MI-136 (4 uM), MI-503 (4 uM) for 120h. Data are shown as mean ± SEM; n = 3.
