# Supplementary material for: Targeting MLL Methyltransferases Enhances the Antitumor Effects of PI3K Inhibition in Hormone Receptor–positive Breast Cancer
Source: Cancer Res Commun. 2022 Dec 6;2(12):1569–78. doi: 10.1158/2767-9764.CRC-22-0158 (PMC10036132; doi:10.1158/2767-9764.CRC-22-0158)
Supplement: Figure S5 — shows that combined PI3K and MLL inhibition provides therapeutic benefit in vitro and in vivo [file crc-22-0158-s05.docx]

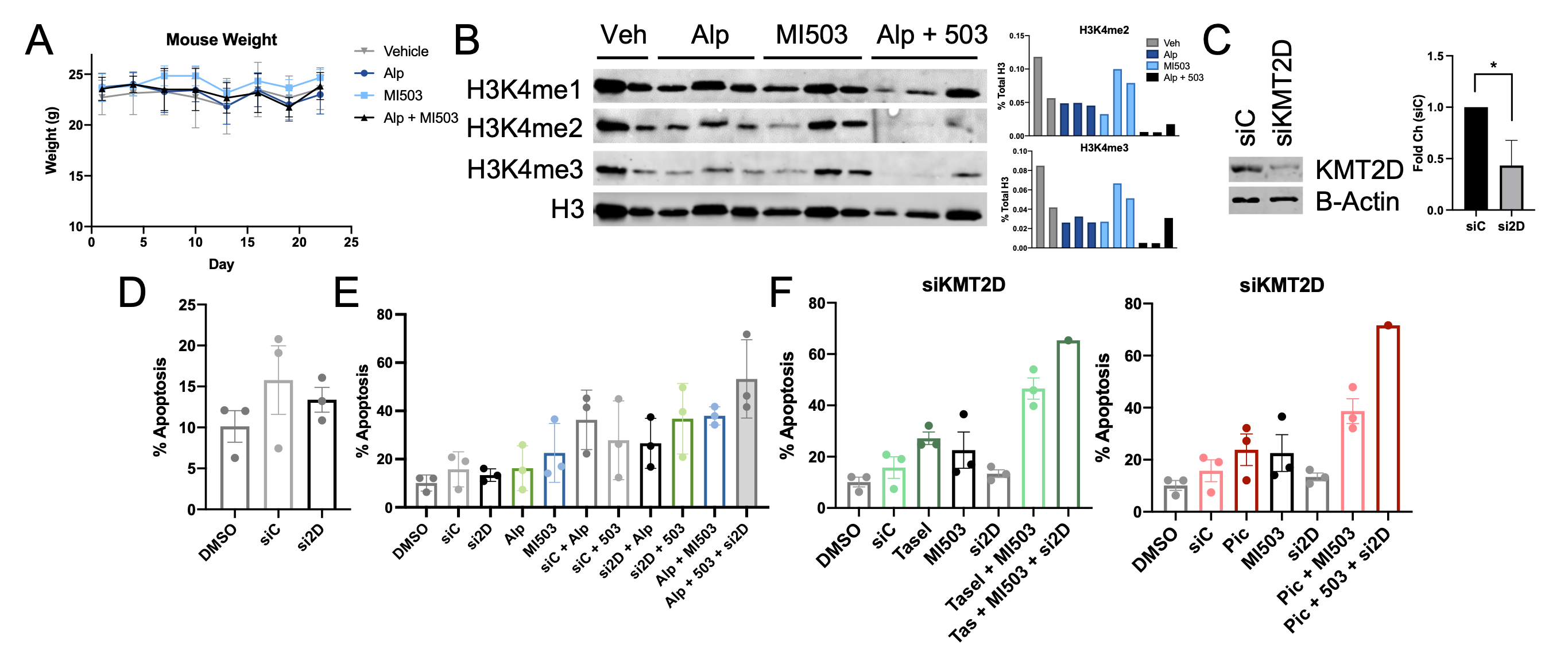


**Supplementary** **Figure 5: Combined MLL and PI3K inhibition provides therapeutic benefit in *in vivo* and *in vitro* models of breast cancer.** (A) Mouse weight with once-daily treatment with alpelisib (45 mg/kg, gavage), MI-503 (30 mg/kg, IP), or the combination. (B) Lysates prepared from tumors isolated from mice in (A) and immunoblotted with the indicated antibodies. (C) MCF7 cells reverse transfected with siControl or siKMT2D for 120h followed by lysate preparation. Lysates were immunoblotted for the indicated antibodies. Immunoblot, left; quantification, right. *n* = 3 independent experiments; *, p < 0.05. (D), (E), and (F) Annexin V staining in MCF7 cells transfected with MLL4/KMT2D or control siRNA and treated with DMSO, alpelisib (1 uM), MI-503 (4 uM), taselisib (1 uM), pictilisib (1 uM) or the combination(s) for 120h. Results shown are representative of at least 3 independent experiments. Data are shown as mean ± SEM.
