# Supplementary material for: Targeting MLL Methyltransferases Enhances the Antitumor Effects of PI3K Inhibition in Hormone Receptor–positive Breast Cancer
Source: Cancer Res Commun. 2022 Dec 6;2(12):1569–78. doi: 10.1158/2767-9764.CRC-22-0158 (PMC10036132; doi:10.1158/2767-9764.CRC-22-0158)
Supplement: Supplementary Table ST1 — Supplementary Table 1 shows p values for IHC staining that corresponds to the graphs shown in Figure 5D [file crc-22-0158-s06.docx]

**Supplementary Table 1: Statistical analyses of immunohistochemistry quantification from xenograft studies in Fig 5.** Images quantitated using Aperio ImageScope, with pixel count per field of view (FOV) grouped into three categories: + (low staining), ++ (medium staining), or +++ (high staining).
